# miRNA nanotherapeutics: potential and challenges in respiratory disorders

**DOI:** 10.4155/fmc-2020-0066

**Published:** 2020-04-09

**Authors:** Meenu Mehta, Dinesh K Chellappan, Peter R Wich, Nicole G Hansbro, Philip M Hansbro, Kamal Dua

**Affiliations:** 1 Discipline of Pharmacy, Graduate School of Health, University of Technology Sydney, NSW, 2007, Australia; 2 Centre for Inflammation, Centenary Institute, Sydney, NSW, 2050, Australia; 3 School of Pharmacy, International Medical University, Bukit Jalil 57000, Kuala Lumpur, Malaysia; 4 School of Chemical Engineering, University of New South Wales, Sydney, NSW, 2052, Australia; 5 Centre for NanoMedicine, University of New South Wales, Sydney, NSW, 2052, Australia; 6 School of Life Sciences, Faculty of Science, University of Technology Sydney (UTS), Ultimo, NSW, 2007, Australia; 7 Priority Research Centre for Healthy Lungs, Hunter Medical Research Institute, University of Newcastle, Lot 1 Kookaburra Circuit, New Lambton Heights, Newcastle, NSW, 2305, Australia; 8 School of Pharmaceutical Sciences, Shoolini University, Solan, Himachal Pradesh, 173229, India

**Keywords:** antagomirs, drug delivery, miRNA, nanoparticles, respiratory disorders

Chronic respiratory disorders are among the most common causes of severe illness and death worldwide. Globally, approximately around 4 million people die annually prematurely due to chronic respiratory diseases [1]. miRNAs are small noncoding RNA molecules that are used to suppress protein translation in order to modulate gene expression. Alterations in miRNA abundance can be observed both in the lung tissue and in the inflammatory cells, as regulators of the disease [2,3]. miRNAs are therefore considered as potential biomarkers and new therapeutic targets for respiratory diseases namely, asthma, chronic obstructive pulmonary disease, lung cancer and fibrosis. Several miRNAs have been identified, especially in the let-7 family, miR-10, miR-26, miR-30, miR-34, miR-99, miR-122, miR-124, miR-122, miR-140, miR-145, myR-146, miR-190, miR-192, miR-219, miR-222 and miR-223 [4], which downregulate their expressions during respiratory disorders. Nevertheless, there is an emerging need to resolve numerous physicochemical and biological challenges associated with miRNAs, particularly, off-target effects, in order to obtain an effective *in vivo* delivery. This commentary, in particular, addresses the challenges associated with the use of miRNAs and the advantages of nonviral methods of delivery in respiratory diseases.

## Therapeutic challenges associated with miRNA

Several biological and physicochemical factors are associated that minimize the delivery of miRNA to the target cells. These include low plasma half-life: in addition to a lack of stability in biological systems and fluids, primarily blood, the short-lived nature of their gene silencing effects is one of the principal obstacles in the use of miRNA. These are enzymatically degraded by cellular nucleases and quickly removed through the kidneys due to their low molecular weights. Studies show that naked miRNA quickly degrades in plasma having half-life of seconds to 30 min *in vivo* [5]. Immunotoxicity: the systemic miRNA delivery, like other types of nuclear acids, stimulates the innate immune system resulting in unintended toxicity and severe adverse effects [6]. Secretion of inflammatory cytokines and Type I interferons by Toll-like receptors (TLRs) result from the systemic administration of miRNA duplexes. These TLR sensor molecules (dsRNA) stimulate both the cellular endosomal and lysosomal interferon Type I pathways, and subsequently, induce the development of cytokine in terms of its structure, sequence and deliveries. TLRs bound by miRNA may also be neurotoxic [7]. Off-target effects: previously, miRNA therapeutics were considered highly specific in their action, but later on studies demonstrated that they are less specific than formerly assumed. They not only bind to complementary sequences but also to similar sequences that lead to show unwanted phenotypes, adverse side effects and sometimes completely negating the therapeutic effect of miRNA. Cell membrane barriers: the naked miRNAs are not free to disperse across the cell membrane. This is because of their comparatively larger molecular weight and polyanionic nature [8].

## MiRNA inhibitors & nanotechnology: a symbiotic association

miRNA inhibitors/antagomirs (AMO) are synthetic miRNA antagonists, also known as miRNA silent agents. They are commonly found bound to the miRNA. They mask the target site, which essentially avoids interaction with the miRNA, enabling them to be translated. This approach has the benefit of annulling the likely off-target effects of a wide range of miRNAs [9]. Like AMO, miRNAs are not degraded by this method. Thus, specific functions of the miRNAs remain intact in other genes. Several reported studies have shown a greater potential for AMO such as AMO-106a and AMO-9, in asthma and hyper-reactive steroidal airways in respiratory diseases [10].

Nanotechnology has made significant strides in recent years, in both developing new materials and also with their applications. These advancements have led to the development of new DNA and RNA delivery systems to monitor diseases that can be used, instead of viral vectors [11]. Inorganic varieties namely, gold, silver, calcium phosphate, graphene, quantum dots, iron oxide and silica; organic substances namely, chacosanes, fabric, protein/peptides and aptamers; polymerized nanomaterials may be used in the architectural creation of these non-viral vectors. Nanomaterial-based delivery systems have several advantages over viral vector delivery systems, which may include, having less immune response and design versatility to work in low cytotoxicity areas [12]. For example, proteins, antibodies and carbohydrates could be used for combining nanoparticles in carboxy and amino groups. The cellular absorption of miRNA is increased because of its small size and the probability of mixing cell penetration peptides [13]. Despite these potential benefits, the applications of nanobiotechnology in the respiratory area are still in its infancy and have not yet made its mark in comparison to other disorders. Few attempts which are made in the respiratory area have been discussed below.

## Evidences on drug delivery approaches of miRNA in respiratory disorders

One of the recent attempts made was to deliver miR146a using polymeric nanoparticles for the treatment of chronic obstructive pulmonary disease. Results showed that miR-146a has maintained its gene and protein functional structure. The high concentration of miR-146a-nanoparticles decreased the *IRAK1* target gene expression to 40% [14]. In addition to that, there is another study which had reported that the presence of miR-660 lipid-nanoparticles decreased lung cancer tumor growth by inducing P53-dependent cell cycle arrest in low doses when compared with the controls [15]. McKiernan *et al.* prepared nanoparticles of miR-126 with cationic polymers and demonstrated their ability to promote the incorporation of miRNA into the CFBE41o cells (the human F508del transmembrane epithelial bronchial regulator for cystic fibrosis), thus significantly decreasing the expression of *TOM1* [16]. Neutral lipid delivery systems have been developed to minimize the adverse impacts and nonspecific interactions of cationic particles. In a non-small-cell lung cancer mouse model for efficacy, a neutral fat emulsion was compared and correlated with synthetic miRNA-34a and let-7 [17]. When compared with cationic lipoplexes, the systemic administration of the neutral lipid particles demonstrated lesser hepatic aggregation, and more uniformly distributed pattern in other tissues such as the lungs. Treatment with the miRNA imitation caused a decrease in the tumor size and an increase in the tissue level of miR-34a [18]. Shi *et al.* developed a codelivery system that comprised of solid lipid nanoparticles enclosing miRNA-34a and paclitaxel (PTX) for synergistic effect to target lung cancer cells. The drugs administered co-operatively and more effectively blocked the metastatic production of BF10-CD44^+^ originating in the lung. In addition, this approach also decreased the doses of PTX, thereby, reducing the side effects associated with PTX alone [19]. A similar study was conducted by Song *et al.* in 2020, which also encapsulated myricetin and siRNA in mesoporous silica nanoparticles and showed its synergistic effect to suppress tumor growth in lung cancer cells with lesser side effects [20].

## Conclusion

The expanded and in-depth research in understanding the roles of miRNA opens up new possibilities for pulmonary disorders. The blend of miRNA with nanotechnology paves the way to achieve enhanced cellular uptake, endosomal escape and improved bioavailability. However, there are several challenges related with the unknown structure and stoichiometry of polymers along with the accumulation of nanoparticles in normal organs. Other key issues awaiting further exploration include effective gene silencing effect with absolute safety and the challenge to increase the delivery of the target substances to the organ of interest or even the cell of interest alone.
